# Adaptation and validation of a Korean version of the speaking up about patient safety questionnaire (KSUPS-Q)

**DOI:** 10.1186/s12912-024-01891-3

**Published:** 2024-04-29

**Authors:** Shinae Ahn, Da Eun Kim

**Affiliations:** 1 Department of Nursing, Wonkwang University, Jeonbuk, Republic of Korea; 2 College of Nursing and Research Institute of Nursing Innovation, Kyungpook National University, Daegu, Republic of Korea

**Keywords:** Speaking up, Patient safety, Communication, Nurses, Psychometrics

## Abstract

**Background:**

Speaking up by healthcare providers is an essential assertive communication strategy for ensuring patient safety and preventing incidents. However, more is needed to know about speaking up and instruments to assess it in the Korean context. Therefore, we assessed the psychometric properties of the Korean version of the Speaking Up about Patient Safety Questionnaire (KSUPS-Q) for measuring speaking up-related behavior and climate among nurses.

**Methods:**

The translation and adaptation process followed the guidelines of the International Society for Pharmacoeconomics and Outcomes Research and the World Health Organization. Content validity was assessed by a six-member expert panel using the content validity index. In total, 314 nurses participated in an online survey to examine the psychometric properties. Internal consistencies were tested using Cronbach’s alpha and McDonald’s omega. Confirmatory factor analyses were conducted to examine the subscales’ construct. The convergent validity of the speaking up-related climate scale was assessed by testing correlations with teamwork and safety climate domains of the Safety Attitudes Questionnaire. In addition, we investigated the convergent validity of the speaking up-related behavior scale by examining its correlation with the climate scale.

**Results:**

The reliability of the 11-item behavior scale was satisfactory. Confirmatory factor analysis confirmed that a three-subscale model (perceived concerns, withholding voice, and speaking up) was appropriate (CFI = 0.98, TLI = 0.98, and SRMR = 0.05). Furthermore, the 11-item climate scale demonstrated satisfactory internal consistency. A three-subscale model (psychological safety, encouraging environment, and resignation) was confirmed (CFI = 0.98, TLI = 0.97, and SRMR = 0.05). The convergent validity of the climate scale was verified based on correlations with the teamwork (*r* = 0.68, *p* < 0.001) and safety climate (*r* = 0.68, *p* < 0.001) domains of the Safety Attitudes Questionnaire. In addition, speaking up-related behavior and climate showed a significant association, indicating that the behavior scale is conceptually valid.

**Conclusions:**

This study demonstrates that the KSUPS-Q is a valid and reliable instrument in Korea. This instrument can help nurse managers simultaneously monitor the behavior and climate of their organizations and evaluate the outcomes of interventions to enhance speaking up. Future research is needed to explore diverse factors contributing to speaking up, including clinical roles, team relationships, and supportive culture, to identify areas requiring further improvement.

**Supplementary Information:**

The online version contains supplementary material available at 10.1186/s12912-024-01891-3.

## Background

Since the publication of *To Err is Human* by the Institute of Medicine [1], patient safety has emerged as a chief component of healthcare quality and a global concern. Despite ongoing efforts to improve patient safety, one in every 10 patients still experiences harm during hospital care [2]. When healthcare providers (HCPs) recognize a potential safety problem, open communication within the healthcare team and stating an opinion before the error results in harm to the patient is crucial for safe care [3–5]. The patient safety principle requires everyone, regardless of hierarchy, to take responsibility and have a voice in raising related safety concerns [6]. Thus, speaking up about patient safety concerns is increasingly acknowledged as an important way to reduce risks [7].

Speaking up refers to assertive communication within healthcare teams, involving immediate action through asking questions, expressing opinions, or exchanging information to address patient safety concerns [3, 8]. Speaking up contributes to the prevention of patient safety incidents (e.g., medication errors, infections, and wrong-site surgeries) and can have an immediate preventive effect on human errors (e.g., failure to follow standards, missed diagnosis) [3, 7]. For example, when an HCP fails to follow hand hygiene protocols, a coworker who speaks up can provide direct and real-time feedback to prevent infections. However, HCPs frequently choose not to speak up owing to various personal, contextual, and organizational factors, including fear of negative feedback, retaliation, presence of patients or relatives, and professional hierarchy [9, 10]. Therefore, speaking up for patient safety requires not only personal communication skills and intentions but also a supportive organizational climate that encourages nurses and other HCPs to report safety concerns.

In recent years, there have been several efforts to assess speaking up for patient safety. Some studies have attempted to measure speaking up using a specific dimension of entire instruments, such as the error reporting dimension of the safety climate instruments [11–13]. However, speaking up focuses more on the preventive effect of human errors [3], while reporting incidents focuses on the occurrence and response to errors [6]. Therefore, the items did not systematically address HCPs’ speaking up behaviors [11]. In addition, a similar concept, a promotive and prohibitive voice scale, was used to measure nurses’ speaking up behavior [14]. Since speaking up and promotive and prohibitive voice are distinct concepts, they may not be adequate to measure using this existing instrument. Thus, developing a single instrument combining climate and behavior is necessary to assess nurses’ speaking up comprehensively.

Survey instruments are the most widely used methods for assessing speaking up. This method allows healthcare organizations to assess and evaluate essential aspects of speaking up to identify educational and organizational needs [11, 15]. It can also compare speaking behavior and climate across time and countries [11, 15]. Meanwhile, prospective observational methods have been used to measure speaking up behavior under simulated or actual general anesthesia [16, 17]. In observation studies, speaking up is measured by the level of speaking up as the time spent, or event-based behavior coding, comprising content, form, and reaction to speaking up. However, it did not measure the degree of withholding in which participants were concerned but remained silent [16]. Although the decision to withhold a HCP’s voice is not an action and cannot be easily observed directly [18], whether a HCP speaks up or withholds his or her voice is essential to measure speaking up behavior [14]. Recently, the scenario approach has been used as a survey method to provide respondents with descriptions of real-life situations, which can minimize personal interpretative variation [19]. A study examined the likelihood of speaking up by presenting vignettes describing hypothetical clinical situations in which a HCP makes an error in patient care [20]. Presenting a typical situation in the vignette allows participants to consider safety concerns in their clinical context and makes their answers less affected by differences from their past experiences or imagined situations [4, 15]. Thus, the scenario approach enables one to measure anticipated behaviors in specific situations using survey questionnaires [15].

Validated instruments help identify factors influencing assertive communication and measure behavior changes, which can be leveraged to promote speaking up. The Speaking Up about Patient Safety Questionnaire (SUPS-Q) is one of the most popular instruments, and it is a self-report scale assessing HCPs’ behaviors, experiences, and perceptions related to speaking up [15]. The SUPS-Q has proven to be an appropriate instrument in terms of its psychometric properties and has been used in various clinical settings in Switzerland and Austria, such as acute care hospitals, pediatric hospitals, psychiatric hospitals, and rehabilitation clinics [4, 15, 21, 22]. The SUPS-Q is a short questionnaire consisting of two scales—speaking up-related behavior and speaking up-related climate—each containing 11 items across three subscales. In addition, the behavior domain includes one item for barriers toward speaking up and a vignette describing a hypothetical situation in which patient safety is jeopardized [15, 22].

Despite the growing importance of speaking up for patient safety, little is known about instruments to assess speaking up in Korea. Considering the safe care process for patients, exploring how HCPs’ speaking up-related behavior relates to their perceptions of their organizations’ speaking up climate is critical in developing assertive communication strategies for reducing risks. Using a validated tool, such as the SUPS-Q, speaking up-related behavior and climate can be investigated simultaneously, and the relationship between the two scales can be identified. However, the psychometric properties of the SUPS-Q have not been verified in the Korean context. It is necessary to ensure the psychometrics of the translated version in the cultural context when using a tool developed in another language [23]. Therefore, we assessed the psychometric properties of the Korean-language version of the SUPS-Q for use in Korean hospital settings, describing the current status of speaking up-related behavior and climate.

## Methods

### Design

This study used a cross-sectional survey design to assess the psychometric properties of the Korean version of the SUPS-Q (KSUPS-Q).

### Sample, setting, and data collection

The participants were clinical nurses who had worked in a hospital-level medical institution for over a month. Convenience sampling was used to recruit participants by distributing a link to an online questionnaire through blog posts, non-profit nursing organizations, and social media platforms. Participants were informed about the study’s aims and methods, and they completed the questionnaires anonymously. The sample size requirements for confirmatory factor analysis (CFA) were determined based on recommendations of ratios of 5–20 cases per item [24], and at least 200 participants for structural equation modelling [25]. Data were collected from August to September 2022. Considering dropout rates in online surveys, a sufficient number of participants were recruited to meet the recommendations for sample size. A total of 315 nurses participated in this study, but one who did not meet the inclusion criteria was excluded. Thus, 314 participants were included in the analysis.

### Measures

#### Speaking up

We used the SUPS-Q, which consists of two domains: speaking up-related behavior and speaking up-related climate. First, the speaking up-related behavior scale consists of 11 items across three factors (perceived concerns, withholding voice, and speaking up) using a 5-point Likert scale ranging from never (0 times) to very often (more than 10 times in the last four weeks). The principal component analysis confirmed the three factors of the behavior scale (total variance explained by the three factors = 65%) [15]. Cronbach’s alphas for perceived concerns, withholding voice, and speaking up on the original scale were 0.73, 0.76, and 0.85, respectively [15]. Additionally, the behavior domain included an item covering barriers toward speaking up and a vignette for anticipated behaviors in a hypothetical situation. The item was one multiple choice question with six options assessing self-perceived barriers in raising patient safety concerns (yes/no). The vignette describing a standardized hypothetical situation (i.e., missed hand hygiene) assessed participants’ anticipated behaviors with four items consisting of perceived realism of the situation, risk of harm to patients, the likelihood of speaking up, and their discomfort with speaking up on a 7-point Likert scale ranging from 1 (not at all) to 7 (very).

Second, the speaking up-related climate was assessed using 11 items across three factors (psychological safety for speaking up, encouraging environment for speaking up, and resignation toward speaking up) using a 7-point Likert scale ranging from 1 (strongly disagree) to 7 (strongly agree) [10, 15, 21]. The principal component analysis confirmed the three factors of the speaking up-related climate scale (total variance explained by the three factors = 60%) [15]. Cronbach’s alphas for psychological safety, encouraging environment, and resignation on the original scale were 0.84, 0.74, and 0.73, respectively [15].

#### Teamwork and safety climate

We used the teamwork and safety climate domains of the Safety Attitudes Questionnaire-Korean version (SAQ-K) [26] to assess the convergent validity of the speaking up-related climate scale.

Responses to 11 items (five items for teamwork climate and six for safety climate) were recorded on a 5-point Likert scale ranging from 1 (strongly disagree) to 5 (strongly agree). Higher mean scale scores indicated more positive perceptions of teamwork and safety climate at the workplace. Cronbach’s alphas for teamwork and safety climate on the original scale were 0.84 and 0.84, respectively [26].

#### General characteristics of participants

Participants’ general characteristics included age, sex, educational level, type of hospital and medical department, job position (staff nurse, charge nurse, or head nurse), job tenure, duration of employment in the present hospital, mean working hours per work shift, experience in patient safety tasks, patient safety education, and patient safety incidents.

The type of hospital was assessed according to the Korean Medical Service Act: hospital, general hospital, advanced general hospital, long-term care hospital, or others [27]. A hospital should have 30 or more beds to provide inpatient medical services. General hospitals have 100 beds and at least seven to nine specialized departments (e.g., internal medicine, general surgery, and pediatrics). Advanced general hospitals are general hospitals designated by the Minister of Health and Welfare to provide highly specialized medical services.

### Translation and adaptation process

We employed a combination of the International Society for Pharmacoeconomics and Outcomes Research (ISPOR) guidelines [28] and the World Health Organization (WHO) guidelines [29]. In addition to ISPOR guidelines, the WHO recommends an expert panel review to identify and resolve inadequate expressions of the translation. Permission to use the questionnaire was obtained from the developers of the SUPS-Q. The SUPS-Q was reviewed by an expert committee comprising two nursing professors and a hospital nurse. The expert committee independently translated the English version of the SUPS-Q. The preliminary version was back translated into English by two other translators who were not involved in the original translation. The clarity and readability of the items were tested by five nurses providing direct care in hospitals; minor modifications in wording were made based on their feedback. The final items were validated through content validity testing for appropriateness and cultural relevance by six expert panel members, including clinical experts in patient safety and nursing professors. They reviewed the translated versions based on the cross-cultural adaptation guidelines considering four aspects: (1) semantic equivalence, (2) idiomatic equivalence, (3) experiential equivalence, and (4) conceptual equivalence [30]. The expert panel discussed ambiguities and discrepancies in a consensus session and agreed on the pre-final translated version. The panel rated each tool on a 4-point scale (not relevant to highly relevant). The item-level content validity index (I-CVI) was calculated as the number of experts who provided ratings of 3 or 4, and the scale CVI (S-CVI) was calculated by computing the mean of the I-CVI scores. A CVI score > 0.88 indicates excellent content validity, and the S-CVI score is required to be > 0.78 if the total number of experts is more than six [31]. The I-CVI scores ranged from 0.83 to 1.00 for both the behavior and climate scales, and their S-CVI scores were 0.98 and 0.97, respectively. All I-CVI and S-CVI scores were 1.00 for barriers toward speaking up and anticipated behaviors in a hypothetical situation, indicating excellent content validity.

No items were deleted or changed from the original questionnaire. We included examples alongside items requiring additional explanation for better understanding and error definitions to ensure clarity, which were derived from expert reviews. As the participants might have been unfamiliar with the concept of “speaking up” in the Korean context, we presented examples of clinical situations wherein patient safety could be threatened and HCPs needed to speak up (e.g., poor hand hygiene, missed patient identification before injections, and improper sterile technique). The term “patient safety incident,” which encompasses near misses, adverse events, and sentinel events, is often used interchangeably with “error” in Korea’s healthcare system. Therefore, based on feedback from the expert panel, we defined the term “error” in the original instrument as a “near miss” and provided the definitions below the survey question to ensure clarity. In addition, as “shift supervisors” is an uncommon expression in Korean hospitals, we added “charge nurse/head nurse” to the relevant items. Seven clinical nurses performed the cognitive debriefing interviews to ensure comprehensibility and time to complete the questionnaire (15–20 min). They were asked to suggest alternative expressions for items they did not understand. After minor changes to their comments, the KSUPS-Q was created for psychometric evaluation.

### Statistical analyses

Descriptive analyses were conducted to examine the frequency, percentage, mean, and standard deviation (SD) of the participants’ general characteristics and questionnaire items. On the speaking up-related climate scale, negatively worded items (9 to 11) are reverse-coded for the total score.

This study examined internal consistency reliabilities for psychometric testing using Cronbach’s alpha and McDonald’s omega (Ω). Cronbach’s alpha is the most widely used and popular method for reliability. Since Cronbach’s alpha is based on the tau-equivalent measurement model, the assumptions of the tau-equivalence (e.g., equal factor loadings of all items) should be met for the alpha coefficient to be equivalent to the reliability coefficient [32]. However, since these assumptions are rarely realistic in practice for psychological scales, it is recommended to use alternative indicators recently. Omega has been referred to as a more sensible index of internal consistency reliability when compared to Cronbach’s alpha and other indexes [33]. Omega shows less risk of overestimation or underestimation of reliability [33]. The Cronbach’s alpha and McDonald’s omega coefficients above 0.7 are acceptable internal consistency [34, 35].

To examine the construct validity of the behavior and climate scales, CFA was conducted to investigate whether the factor structure of the original SUPS-Q [15] could be confirmed for the Korean data. Prior to performing the CFA, multivariate normality was assessed using Mardia’s test to confirm the suitability of the dataset for factor analysis. As both the behavior and climate scales did not satisfy the assumption of multivariate normality, weighted least squares mean and variance-adjusted estimation, which can be used with ordinal item distributions without assuming multivariate normality, were performed [36]. Model fit indices included the comparative fit index (CFI) and Tucker–Lewis index (TLI) ≥ 0.90, and the standardized root mean square residual (SRMR) ≤ 0.08 [37].

Convergent validity indicates a correspondence between theoretically similar concepts [38]. High correlation coefficients with other validated instruments demonstrate convergent validity. The convergent validity of the speaking up-related behavior scale was confirmed by examining its correlation with speaking up-related climate scale. In addition, we examined the correlation between four items for anticipated behaviors in a hypothetical situation and the speaking up-related behavior scale to identify the convergent validity of the four items. The convergent validity of the speaking up-related climate scale was determined by examining its correlation with the two climate domains of the SAQ-K (teamwork and safety). The SAQ is widely used to measure patient safety culture, and consists of six subscales including safety and teamwork climate [26]. HCPs’ speaking up is influenced by organizational safety climate, which covers various aspects such as psychological safety and teamwork [21]. Teamwork climate would influence speaking up, which refers to assertive communication. Communication is an essential skill for team performance and one of the teamwork components that ensures safe care [39]. Thus, based on previous studies [11, 21, 26, 39], we hypothesized that safety climate and teamwork climate scores would be significantly more positive in environments where HCPs can speak up about safety issues. The minimum criterion for acceptable convergent validity was *r* ≥ 0.3 [40]. Content validities of all items were examined using the CVI in the translation and adaptation process.

In addition, independent t-tests were performed to identify differences in speaking up-related behavior, anticipated behaviors in a hypothetical situation, and speaking up-related climate according to patient safety task experience. Based on the literature [3, 41], HCPs’ role identification is one of the factors influencing speaking up behavior [3]; nurses with clear role identification assigned to patient safety tasks were able to raise safety concerns more easily [41]. For example, when nurses were designated as clinical champions (e.g., hand hygiene or fall prevention activities) for patient safety in their organizations, their task was to monitor and provide feedback to other HCPs about patient safety performance, which helped them speak up [41]. We expected speaking up scores to be more positive in the group with experience in patient safety tasks.

Omega coefficient calculation and CFA were performed using Mplus ver. 8.8 (Muthén & Muthén, Los Angeles, CA, USA). All other analyses were performed using SPSS Statistics ver. 26.0 (IBM Corp., Armonk, NY, USA).

## Results

### General characteristics of participants

The participants’ mean age was 33.71 years (SD = 7.59), and 89.5% were female (Table 1). Approximately 81.2% were staff nurses, 13.4% were charge nurses, and 5.4% were head nurses. Approximately 42.7% had experience in patient safety tasks, and 83.8% had received patient safety education at least once within one year.

**Table 1:** General characteristics of the participants (*N* = 314)

Variables	Categories	n (%)	Mean (SD)
Age (years)			33.71 (7.59)
Female		281 (89.5)	
Educational level	College diploma	35 (11.1)	
	Bachelor’s degree	192 (61.1)	
	Pursuing a master’s program or master’s degree	64 (20.4)	
	Pursuing a doctoral program or doctoral degree	23 (7.4)	
Type of hospital	Hospital	29 (9.2)	
	General hospital	81 (25.8)	
	Advanced general hospital	190 (60.5)	
	Long-term care hospital	12 (3.8)	
	Others	2 (0.6)	
Medical department	Internal medicine	59 (18.8)	
	Surgery	68 (21.7)	
	Intensive care unit	49 (15.6)	
	Operating room, recovery room	47 (15.0)	
	Emergency room	29 (9.2)	
	Outpatient services	31 (9.9)	
	Other areas	31 (9.9)	
Position	Staff nurse	255 (81.2)	
	Charge nurse	42 (13.4)	
	Head nurse	17 (5.4)	
Job tenure (months)			109.61 (88.37)
Duration of employment in the present hospital (months)			49.72 (53.97)
Mean working hours per work shift			8.77 (0.96)
Experience in patient safety tasks	Yes	134 (42.7)	
Received patient safety education	Yes	263 (83.8)	
Number of sessions on patient safety education (*n* = 263)			2.16 (1.72)
Experienced patient safety incidents	Yes	245 (78.0)	

*SD* standard deviation

### Speaking up-related behavior assessment

#### Speaking up-related behavior scale

**Fig. 1:**
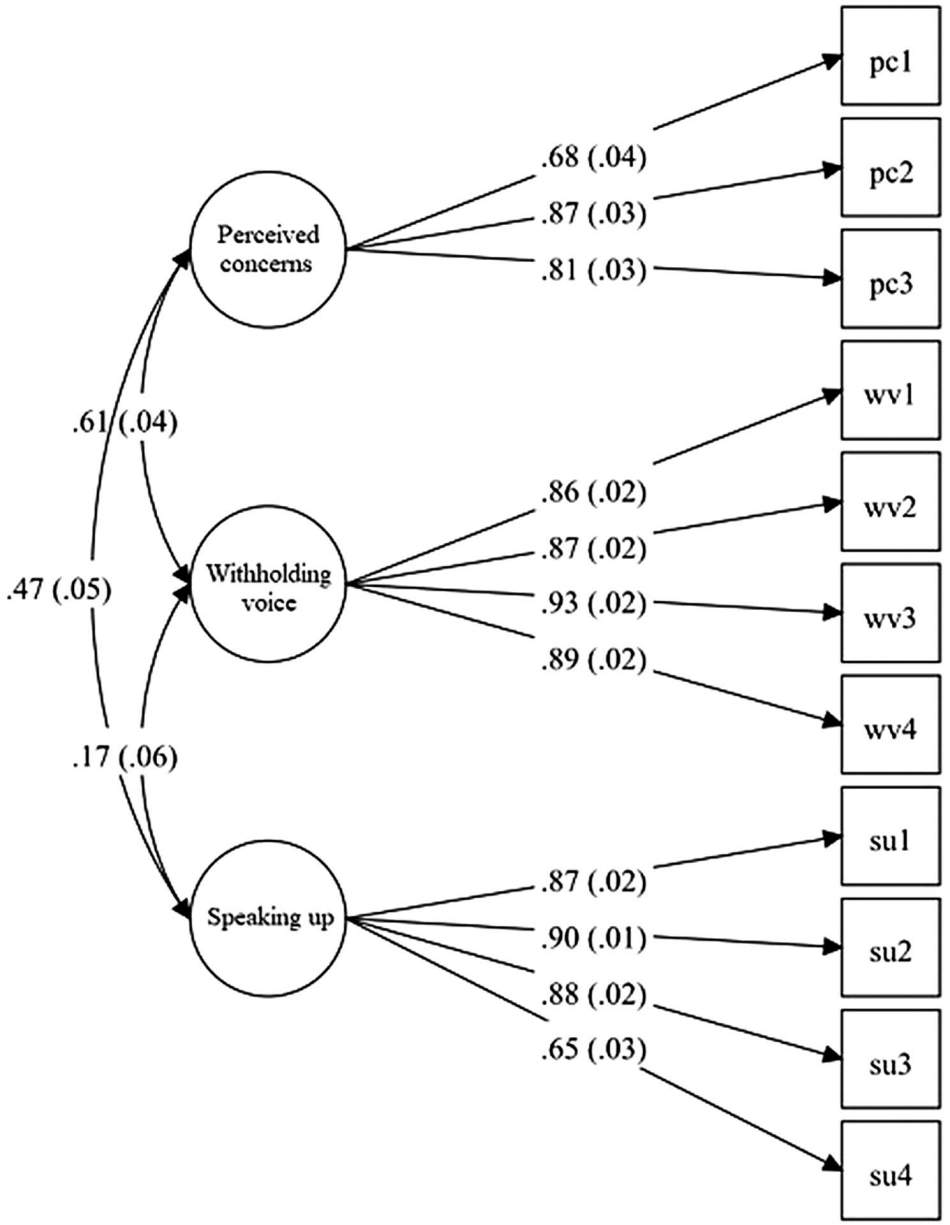
Three-factor model of the speaking up-related behavior scale *PC* perceived concerns, *SU* speaking up, *WV* withholding voice *Note*. The numbers shown in the figure from left to right are standardized: (1) correlation coefficients and standard errors among three factors, (2) factor loadings and standard errors, all of which are significant (*p* < 0.001)

The mean score for “perceived concern” was 2.26 (SD = 0.75), “withholding voice” was 1.73 (SD = 0.82), and “speaking up” was 2.45 (SD = 0.91; Table 2). The reliabilities of “perceived concerns” (Cronbach’s alpha = 0.75, omega = 0.83), “withholding voice” (Cronbach’s alpha = 0.89, omega = 0.94), and “speaking up” (Cronbach’s alpha = 0.87, omega = 0.90) were acceptable. CFA indicated that the three-factor model was appropriate and fit adequately (CFI = 0.98, TLI = 0.98, and SRMR = 0.05). Factor loadings in the CFA model are shown in Fig. 1.

**Table 2:** Mean and SD of the speaking up-related behavior scale according to experience in patient safety tasks

In everyday work, it sometimes happens that things go wrong and risks to patients arise. This could be as a result of medication error, poor hand hygiene, or missing documentation. Over the past 4 weeks, how often…		Total (*N* = 314)	Experience in patient safety tasks		
			Yes (*n* = 134)	No (*n* = 180)	t (*p*)
		Mean (SD)	Mean (SD)	Mean (SD)	
**Perceived concerns** (Cronbach’s alpha = 0.75, omega = 0.83)		2.26 (0.75)	2.42 (0.83)	2.14 (0.67)	-3.29 (0.001)
PC1	Have you had specific concerns about patient safety?	2.57 (0.97)	2.75 (1.04)	2.44 (0.90)	-2.87 (0.004)
PC2	Have you observed an error which—if uncaptured—could be harmful to patients?	2.16 (0.82)	2.31 (0.90)	2.06 (0.73)	-2.63 (0.009)
PC3	Have you noticed that your workplace colleagues have not followed important patient safety rules, intentionally or unintentionally?	2.04 (0.97)	2.21 (1.03)	1.91 (0.90)	-2.72 (0.007)
**Withholding voice** (Cronbach’s alpha = 0.89, omega = 0.94)		1.73 (0.82)	1.74 (0.87)	1.71 (0.78)	-0.34 (0.367)
WV1	Did you choose not to bring up your specific concerns about patient safety?	1.75 (0.97)	1.81 (1.03)	1.70 (0.92)	-0.96 (0.337)
WV2	Did you keep ideas for improving patient safety in your unit to yourself?	1.80 (0.93)	1.77 (0.93)	1.82 (0.94)	0.45 (0.653)
WV3	Did you remain silent when you had information that might have prevented a safety incident in your unit?	1.65 (0.96)	1.66 (0.96)	1.64 (0.96)	-0.18 (0.857)
WV4	Did you not address a colleague (doctors and/or nurses) if he/she did not follow important patient safety rules, intentionally or unintentionally?	1.71 (0.91)	1.74 (0.97)	1.69 (0.87)	-0.48 (0.632)
**Speaking up** (Cronbach’s alpha = 0.87, omega = 0.90)		2.45 (0.91)	2.62 (0.93)	2.33 (0.88)	-2.82 (0.005)
SU1	Did you bring up specific concerns about patient safety?	2.65 (1.10)	2.85 (1.07)	2.50 (1.11)	-2.82 (0.005)
SU2	Did you address an error which—if uncaptured—could be harmful for patients?	2.55 (1.09)	2.69 (1.09)	2.45 (1.08)	-1.98 (0.049)
SU3	Did you address a colleague (doctors and/or nurses) when he/she did not follow important patient safety rules, intentionally or unintentionally?	2.39 (1.05)	2.54 (1.09)	2.27 (1.01)	-2.23 (0.027)
SU4	Did you prevent an incident from occurring as a consequence of bringing up specific concerns about patient safety?	2.22 (1.07)	2.39 (1.10)	2.09 (1.04)	-2.47 (0.014)

*PC* perceived concerns, *SD* standard deviation, *SU* speaking up, *WV* withholding voice

#### Reported barriers toward speaking up

**Fig. 2:**
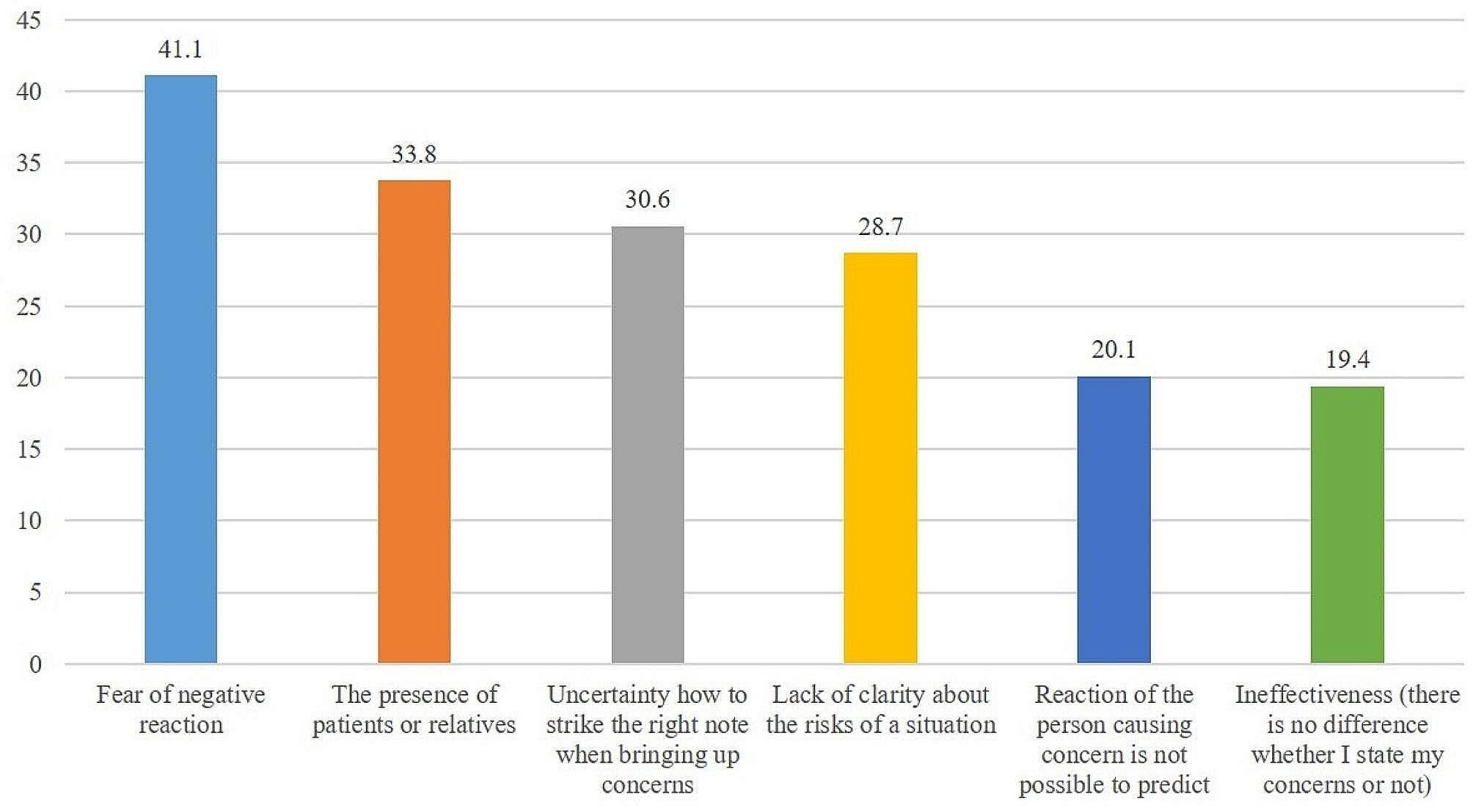
Frequencies (%) of reported barriers toward speaking up

Figure 2 shows the frequencies of the reported barriers toward speaking up. Fear of negative reactions was the most frequently reported barrier (*n* = 129, 41.1%). Additionally, approximately one-third of the participants answered that it was difficult to speak up owing to the presence of patients or their relatives (*n* = 106, 33.8%).

#### Anticipated behaviors in a hypothetical situation

Table 3 presents the results of the four-item vignette to measure anticipated behaviors in the hypothetical situation. The item with the highest mean score was “If nobody acts, how dangerous do you think this situation is for the patient?” (mean = 5.63, SD = 0.95), followed by “How realistic is this situation?” (mean = 5.24, SD = 1.40). The detailed results of the correlation analysis between the four items and the speaking up-related behavior scale are displayed in Additional File 1. All four items were related to “withholding voice.” Furthermore, the “likelihood of speaking up” item was related to “speaking up” (*r* = 0.21, *p* < 0.001).

**Table 3:** Mean and SD for anticipated behavior in a hypothetical situation (Vignette)

You are on a daily round with several doctors and nurses. During the round, the consultant doctor does not wear gloves and/or disinfect their hands before examining the patient’s wound.		Total (*N* = 314)	Experience in patient safety tasks		
			Yes (*n* = 134)	No (*n* = 180)	t (*p*)
		Mean (SD)	Mean (SD)	Mean (SD)	
Realistic	How realistic is this situation? (1 = not at all, 7 = very realistic)	5.24 (1.40)	5.28 (1.49)	5.21 (1.34)	-0.45 (0.651)
Risk of harm	If nobody acts, how dangerous do you think this situation is for the patient? (1 = not dangerous at all, 7 = very dangerous)	5.63 (0.95)	5.78 (0.89)	5.52 (0.98)	-2.44 (0.015)
Likelihood of speaking up	How likely is it that you try to alert the consultant to the missed hand disinfection/gloves (using words or gestures)? (1 = very unlikely, 7 = very likely)	4.44 (1.68)	4.57 (1.71)	4.35 (1.66)	-1.13 (0.258)
Discomfort	Would you feel uncomfortable to instruct the consultant to disinfect their hands/wear gloves? (1 = not at all uncomfortable, 7 = very comfortable)	4.69 (1.60)	4.75 (1.64)	4.65 (1.57)	-0.57 (0.570)

*SD* standard deviation

### Speaking up-related climate assessment

**Fig. 3:**
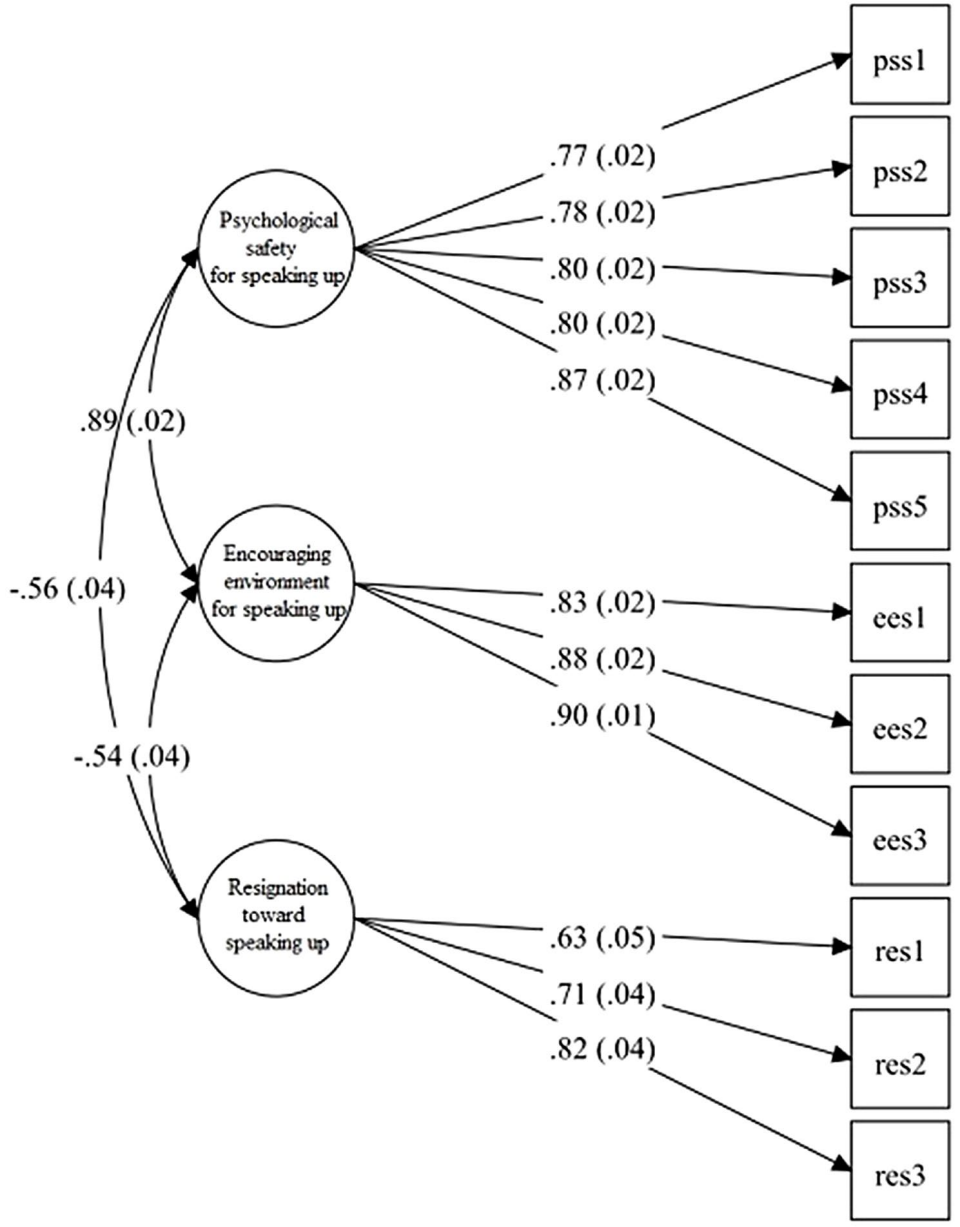
Three-factor model of the speaking up-related climate scale *EES* encouraging environment for speaking up, *PSS* psychological safety for speaking up, *RES* resignation toward speaking up *Note*. The numbers shown in the figure from left to right are standardized: (1) correlation coefficients and standard errors among three factors, (2) factor loadings and standard errors, all of which are significant (*p* < 0.001)

The mean score for “psychological safety for speaking up” was 4.97 (SD = 1.12), “encouraging environment for speaking up” was 4.72 (SD = 1.33), and “resignation toward speaking up” was 3.95 (SD = 1.24; Table 4). The reliability of the whole scale was acceptable (Cronbach’s alpha = 0.90, omega = 0.93). Similarly, “psychological safety for speaking up” (Cronbach’s alpha = 0.88, omega = 0.90), “encouraging environment for speaking up” (Cronbach’s alpha = 0.88, omega = 0.90), and “resignation toward speaking up” (Cronbach’s alpha = 0.67, omega = 0.77) showed acceptable reliability. CFA confirmed that the three-factor model was adequate with an acceptable fit (CFI = 0.98, TLI = 0.97, and SRMR = 0.05). Factor loadings in the CFA model are shown in Fig. 3.

**Table 4:** Mean and SD for the speaking up-related climate scale according to experience in patient safety tasks

Item content	Total (*N* = 314)	Experience in patient safety tasks		
		Yes (*n* = 134)	No (*n* = 180)	t (*p*)
	Mean (SD)	Mean (SD)	Mean (SD)	
**Factor 1. Psychological safety for speaking up** (Cronbach’s alpha = 0.88, omega = 0.90)	4.97 (1.12)	5.00 (1.16)	4.95 (1.10)	-0.38 (0.704)
1. I can rely on my colleagues (doctors and/or nurses) whenever I encounter difficulties in my work.	4.98 (1.42)	5.01 (1.42)	4.97 (1.42)	-0.25 (0.801)
2. I can rely on the shift supervisor (person in charge of a shift: e.g., charge nurse, head nurse) whenever I encounter difficulties in my work.	4.92 (1.44)	4.94 (1.48)	4.91 (1.41)	-0.21 (0.833)
3. The culture in my unit/clinical area makes it easy to speak up about patient safety concerns.	4.85 (1.37)	4.99 (1.40)	4.74 (1.35)	-1.54 (0.124)
4. My colleagues (doctors and/or nurses) react appropriately, when I speak up about my concerns about patient safety.	4.94 (1.33)	4.89 (1.34)	4.98 (1.32)	0.63 (0.530)
5. My shift supervisors (person in charge of a shift: e.g., charge nurse, head nurse) react appropriately, when I speak up about my patient safety concerns.	5.14 (1.31)	5.16 (1.33)	5.13 (1.31)	-0.16 (0.876)
**Factor 2. Encouraging environment for speaking up** (Cronbach’s alpha = 0.88, omega = 0.90)	4.72 (1.33)	4.67 (1.42)	4.76 (1.25)	0.58 (0.560)
6. In my unit/clinical area, I observe others speaking up about their patient safety concerns.	4.71 (1.41)	4.60 (1.49)	4.80 (1.35)	1.26 (0.209)
7. I am encouraged by my colleagues (doctors and/or nurses) to speak up about patient safety concerns.	4.57 (1.54)	4.55 (1.61)	4.58 (1.49)	0.15 (0.885)
8. I am encouraged by my shift supervisor (person in charge during a shift: e.g., charge nurse, head nurse) to speak up about patient safety concerns.	4.88 (1.48)	4.86 (1.64)	4.89 (1.36)	0.21 (0.831)
**Factor 3. Resignation toward speaking up** (Cronbach’s alpha = 0.67, omega = 0.77)	3.95 (1.24)	4.11 (1.26)	3.83 (1.22)	-2.00 (0.047)
9. When I have patient safety concerns, it is difficult to bring them up.^1^	3.83 (1.55)	3.78 (1.62)	3.87 (1.50)	0.47 (0.639)
10. Having to remind staff of the same safety rules again and again is frustrating.^1^	3.86 (1.61)	4.09 (1.65)	3.69 (1.57)	-2.19 (0.029)
11. Sometimes I become discouraged because nothing changes after expressing my patient safety concerns.^1^	4.17 (1.63)	4.47 (1.63)	3.94 (1.60)	-2.86 (0.005)
**Total mean speaking up-related climate score** (Cronbach’s alpha = 0.90, omega = 0.93)	4.65 (1.03)	4.60 (1.07)	4.68 (1.00)	0.67 (0.505)

*SD* standard deviation

^1^Negatively worded items are reverse coded for the total score

We examined the correlation between the two subdomains of the SAQ-K (i.e., teamwork and safety climate) and the speaking up-related climate scale to determine convergent validity. The mean teamwork climate score was 3.33 (possible range: 1 to 5), and the mean safety climate score was 3.41 (possible range: 1 to 5). All subscales of the speaking up-related climate scale were significantly correlated with the teamwork and safety climate domains of the SAQ-K (Table 5). The range of Pearson’s values was − 0.38 to 0.72.

**Table 5:** Correlations between the speaking up-related climate scale and the teamwork and safety climate domains of the Safety Attitudes Questionnaire

Domains	Mean (SD)	Total score	Subscales		
			Psychological safety for speaking up	Encouraging environment for speaking up	Resignation toward speaking up
		r (*p*)			
Teamwork climate	3.33 (0.76)	0.68 (< 0.001)	0.72 (< 0.001)	0.57 (< 0.001)	-0.38 (< 0.001)
Safety climate	3.41 (0.75)	0.68 (< 0.001)	0.67 (< 0.001)	0.64 (< 0.001)	-0.37 (< 0.001)

*SD* standard deviation

### Correlations between speaking up-related behavior and climate scales

We examined the correlations between the speaking up-related behavior and climate scales to determine the convergent validity of the speaking up-related behavior scale. Perceived concerns and withholding voice from the speaking up-related behavior scale were negatively correlated with psychological safety and encouraging environment for speaking up, and positively correlated with resignation toward speaking up (Table 6). Speaking up was correlated with psychological safety and encouraging environment for speaking up. Still, there was no significant correlation between speaking up and a climate of resignation toward speaking up.

**Table 6:** Correlations between speaking up-related behavior and climate

		Speaking up-related behavior		
		Perceived concerns	Withholding voice	Speaking up
		r (*p*)		
Speaking up-related climate	Psychological safety for speaking up	-0.15 (0.009)	-0.31 (< 0.001)	0.12 (0.041)
	Encouraging environment for speaking up	-0.15 (0.007)	-0.39 (< 0.001)	0.12 (0.040)
	Resignation toward speaking up	0.32 (< 0.001)	0.45 (< 0.001)	0.05 (0.361)

### Speaking up-related behavior and climate according to patient safety task experience

Participants with experience in patient safety tasks were more likely to have perceived concerns (t = -3.29, *p* = 0.001) and speak up about patient safety (t = -2.82, *p* = 0.005; Table 2). These participants were also more likely to respond than those without experience that if nobody acted in this hypothetical situation (i.e., missed hand hygiene), it would be dangerous for the patient (t = -2.44, *p* = 0.015; Table 3). Participants with experience in patient safety tasks were more likely to experience resignation toward speaking up about patient safety (t = -2.00, *p* = 0.047; Table 4).

## Discussion

Given that there is increasing evidence that speaking up about patient safety concerns in clinical situations contributes to patient safety, this study examined the psychometric properties of the Korean version of SUPS-Q, which allows for the assessment of speaking up-related behavior and perceived climate. The original SUPS-Q was developed in Switzerland and primarily used in Western cultures, including Switzerland and Austria. As sociocultural contexts can influence HCPs’ expression or withholding of patient safety concerns [42], speaking up-related behaviors and factors influencing them may differ in Western and East Asian cultures. Hence, it is inadequate to assess speaking up in an East Asian cultural context using instruments developed in Western countries without validation processes [43]. Therefore, we adapted the KSUPS-Q using a cultural adaptation process and demonstrated its psychometric properties, including its reliability and validity in Korean hospital settings.

Regarding reliability, Cronbach’s alpha and McDonald’s omega values of the three subscales of the speaking up-related behavior scale showed satisfactory internal consistencies. These results are consistent with a previous study in which the original SUPS-Q was developed in Swiss hospitals, indicating that Cronbach’s alpha for the three subscales was 0.73 to 0.85 [15]. Furthermore, a previous study in an Austrian university hospital showed that these subscales had a satisfactory Cronbach’s alpha of 0.74–0.88 [10]. Regarding the speaking up-related climate scale, the scale and two subscales (psychological safety and encouraging environment) showed satisfactory Cronbach’s alpha and omega coefficients. Meanwhile, Cronbach’s alpha of the other subscale, resignation, was slightly low (0.67), but the omega coefficient was acceptable (0.77). Regarding Cronbach’s alpha, the reliability value could be underestimated if the assumption of tau-equivalence was not met [32]. Furthermore, given that omega outperforms Cronbach’s alpha under violations of tau-equivalence [33], it can be concluded that the speaking up-related behavior and climate scales had acceptable internal consistency.

CFA is used to test hypotheses about the factor structure of data by examining the goodness of fit of the predetermined factor model. The CFA demonstrated the appropriateness of the three-subscale model of the speaking up-related behavior and climate scales in Korean hospital settings. In addition, factor loadings of each item of the behavior and climate scales were 0.65–0.93 and 0.63–0.90, respectively, indicating a satisfactory fit (> 0.5) [44]. Psychological safety and encouraging environment of the climate scale may seem somewhat related concepts, but they have been regarded as distinct concepts [15]. The psychological safety subscale measures more cultural conditions, such as relying on colleagues or supervisors for difficulties at work or perceiving the appropriate response to speaking up about patient safety concerns [15]. Meanwhile, the encouraging environment subscale measures the extent to which respondents are aware of being encouraged by colleagues or supervisors to speak up or observe others speaking up.

This study demonstrated convergent validity of the speaking up-related climate with teamwork and safety climate domains of the SAQ, which means nurses who recognized that their hospital environments are easy to speak up about patient safety concerns were more likely to report high scores for teamwork and safety climate. These two types of climates can have positive influences on the speaking up-related climate. This is because the high quality of teamwork between HCPs supports an environment that allows for assertiveness, which promoted nurses’ speaking up behavior, and organizational commitment to safety creates an encouraging environment for open communication [21, 43]. Therefore, the significant relationship supports the idea that the climate scale is a conceptually valid instrument. In addition, we demonstrated the convergent validity by examining the relationship with speaking up-related climate regarding the speaking up-related behavior scale. This indicates that a supportive climate to speak up is associated with safety-related communication behavior. These results are consistent with a previous study which demonstrated that an encouraging environment for speaking up was associated with a higher frequency of speaking up (OR = 1.25, 95% CI = 1.07–1.47) and lower frequency of withholding voice (OR = 0.82, 95% CI = 0.71–0.95) [21]. The validation study of SUPS-Q also examined correlations between speaking up–related behavior and climate scales [15]. All subscales of the speaking up-related climate scale showed stronger correlations with withholding voice than speaking up [15], which is a consistent finding with our study. It can be assumed that the perceived climate toward speaking up might be more critical for remaining silent than assertive communicative behavior such as speaking up. Thus, it is necessary to identify the factors influencing speaking up and withholding voice using the KSUPS-Q.

We found that nurses in Korean hospitals perceived safety concerns more frequently, remained silent more often, and spoke up less than those in Austrian hospitals [10]. The main barrier to speaking up was fear of negative reactions, which could be an indicator of the hierarchy and authority culture. In a qualitative study, nurses’ speaking up was negatively affected by hierarchical constraints and power dynamics, lack of support, and experiences of being ignored or disrespected [45]. In East Asian cultures, seniority-based hierarchies play a significant role in speaking up, and seniority is determined by age and job longevity [43]. In these cultures, junior staff may not express their concerns to senior colleagues or managers [43], making hierarchy a deciding factor in their silence.

In the present study, nurses’ total scores on the speaking up-related climate scale were lower than those reported in a Swiss study [4]. In Korea, since the enactment of the Patient Safety Act in 2016, various strategies have been implemented to reduce harm and create a patient safety environment in clinical settings [46]. Nevertheless, there are negative dimensions that hinder a safe environment, such as a hierarchical culture and indirect and unclear communication styles [47]. Thus, it is necessary to create a safe and encouraging environment that supports speaking up, and repeatedly perform measurements using a validated instrument to detect changes.

The SUPS-Q is sufficiently sensitive to discriminate between speaking up-related behavioral patterns in different groups [15]. Compared with doctors and HCPs without managerial functions, nurses and HCPs with managerial functions perceived safety concerns in their workplace more frequently [10, 21]. A novel finding of the present study is comparing the degree of speaking up between participants with and without experience in patient safety tasks. Nurses with experience in patient safety tasks were more likely to perceive safety concerns and showed significantly higher levels of speaking up-related behaviors than those without such experience. Speaking up-related behavior must be emphasized in healthcare organizations and demonstrated by leaders [45]. Based on definitions of leadership, leaders can directly or indirectly affect patient safety and quality of care [48]. Leaders can impact quality improvement and safety and create a safety culture by serving as role models, and training employees in the knowledge, skills, and attitudes required for safer care [48]. Several studies have emphasized the importance of team relationships and the attitude of a senior member or team leader in increasing the feeling of safety for speaking up [3]. In this study, nurses with experience in patient safety tasks perceived dangerous situations and may have initiated communication to reduce risks more often because they had a higher level of patient safety awareness. Thus, nurses with experience in patient safety tasks can play an important leadership role and directly or indirectly influence the perception of speaking up.

In this study, although nurses with experience in patient safety were more likely to speak up, they also reported higher levels of resignation. When a nurse raises their voice to speak up about safety threats but other coworkers react negatively, they may feel “frustrated” and like they are “making no change.” In addition, resignation toward speaking up was significantly associated with withholding voice in this study. These findings imply that experiencing negative reactions to speaking up is crucial for predicting future behavior because resignation plays a critical role in the culture of silence, lowering the chances of speaking up [18]. Previous favorable experiences of speaking up to others can enhance speaking up behaviors [41]. Considering that nurses can perceive speaking up as valuable and practical through positive speaking up experiences, creating a supportive organizational culture that respects and responds to other’s opinions about patient safety is necessary.

This study has important implications for creating safe healthcare environments. Previous studies have shown that individual, team, contextual, organizational, and sociocultural factors can affect HCPs’ decision to speak up or remain silent concerning safety issues [43, 49]. An organizational safety climate and culture is crucial to patient safety [3, 43]. A higher level of psychological safety and an encouraging environment are associated with a higher likelihood of frequent speaking up [21]. The speaking up-related climate scale of the KSUPS-Q can be used to assess various levels of personal, team, organizational, and cultural factors. Therefore, the KSUPS-Q can help identify the degree of the speaking up-related climate in Korean hospitals and can be employed in comparative studies with other countries.

A new approach can help to encourage speaking up at diverse levels. In traditional approaches, healthcare managers typically focus on standardizing work practices. However, HCPs can adjust their work to conditions rather than work as imagined [50]. Because the healthcare environment is complex and unpredictable, HCPs interact directly with a hazardous process in daily work [50–52]. There can often be a discrepancy between how everyday work happens (work as done) and how work should be presumed to have occurred (work as imagined) [50, 51]. This gap can lead to safety issues, but we can learn from all the work results, including positive and negative outcomes and everything in between, which is the concept of the Safety-II approach [52]. Therefore, based on the Safety-II approach, healthcare managers should look at many cases of speaking up and things going right in their work unit to achieve acceptable outcomes and try to understand how that happens. The KSUPS-Q can be helpful for nurse managers to repeatedly monitor and measure organizational changes and identify areas requiring further improvement for the quality and safety of patient care.

However, this study had some limitations. First, the data were collected using self-reported questionnaires and were therefore subjective. Second, since the participants were recruited using convenience sampling, the generalizability of the results might be limited. Third, test-retest reliability and discriminant validity were not evaluated. Thus, future research with repeated measures should be conducted to assess test-retest reliability and discriminant validity. In addition, we recommend conducting large-scale studies to determine speaking up-related behavior and climate across various samples and settings.

## Conclusions

This study assessed the psychometric properties of KSUPS-Q in the Korean healthcare context. These findings supported satisfactory validity and reliability of the instrument for nurses in hospital settings. The KSUPS-Q is a short questionnaire systematically measuring speaking up-related behavior and climate aspects. The KSUPS-Q can contribute to investigating personal, team, organizational, and cultural factors, such as clinical roles, team relationships, and supportive culture, that influencing nurses’ willingness to speak up or remain silent in the Korean context. Furthermore, researchers could use this instrument to evaluate outcomes of speaking up-related interventions to enhance patient safety.

### Electronic supplementary material

Below is the link to the electronic supplementary material.

Supplementary Material 1

## Data Availability

All data relevant to the study are included in the article. Data cannot be shared for ethical/privacy reasons.

## Abbreviations

CFA: Confirmatory factor analysis
CFI: Comparative fit index
HCPs: Healthcare providers
I-CVI: Item-level content validity index
KSUPS-Q: Korean version of the Speaking Up about Patient Safety Questionnaire
PCA: Principal component analysis
S-CVI: Scale content validity index
SAQ-K: Safety Attitudes Questionnaire-Korean version
SD: Standard deviation
SRMR: Standardized root mean square residual
SUPS-Q: Speaking Up about Patient Safety Questionnaire
TLI: Tucker–Lewis index
