# Supplementary material for: Adaptation and validation of a Korean version of the speaking up about patient safety questionnaire (KSUPS-Q)
Source: BMC Nurs. 2024 Apr 29;23:293. doi: 10.1186/s12912-024-01891-3 (PMC11057173; doi:10.1186/s12912-024-01891-3)
Supplement: Supplementary file 1 — Supplementary Material 1 [file 12912_2024_1891_MOESM1_ESM.pdf]

Additional file 1. Correlations between anticipated behaviors in a hypothetical situation and speaking up-related behavior

Anticipated behaviors in a hypothetical situation	Speaking up-related behavior		
	Perceived concerns	Withholding voice	Speaking up
	r (*p*)		
**Realistic**	0.13 (0.018)	0.19 (0.001)	-0.09 (0.121)
**Risk of harm**	0.04 (0.494)	-0.14 (0.016)	0.07 (0.193)
**Likelihood of speaking up**	-0.07 (0.210)	-0.26 (<0.001)	0.21 (<0.001)
**Discomfort**	0.21 (<0.001)	0.27 (<0.001)	-0.09 (0.096)
